# Gemcitabine-Induced Lung Injury Masquerading as ARDS in a Patient With Metastatic Renal Medullary Carcinoma: A Case Report

**DOI:** 10.7759/cureus.104939

**Published:** 2026-03-09

**Authors:** Dawit Worku, Alehegn Gelaye, Sara Gezahegn

**Affiliations:** 1 Division of Pulmonary and Critical Care Medicine, Henry Ford Health/Michigan State University College of Human Medicine, Henry Ford Health Providence Hospital, Southfield, USA

**Keywords:** acute respiratory failure, drug-induced lung injury, gemcitabine pulmonary toxicity, noncardiogenic pulmonary edema, renal medullary carcinoma

## Abstract

Gemcitabine is widely used in the treatment of solid tumors, but pulmonary toxicity remains a rare and potentially life-threatening complication. We report the case of a woman with metastatic SMARCB1-deficient renal medullary carcinoma who developed acute hypoxic respiratory failure shortly after receiving combination gemcitabine and carboplatin. She presented with dyspnea, cough, fever, and diffuse pulmonary infiltrates initially attributed to multifocal pneumonia or emerging acute respiratory distress syndrome (ARDS). Respiratory viral testing was positive for rhinovirus, but bacterial cultures remained negative. Cardiac function was preserved, and pulmonary embolism was excluded. Computed tomography demonstrated diffuse lower lobe-predominant ground-glass opacities, patchy airspace disease, and peribronchial thickening with a small pleural effusion, consistent with noncardiogenic pulmonary edema or inflammatory lung injury. The temporal association with chemotherapy, absence of infectious or cardiogenic causes, and rapid clinical response to corticosteroids supported the diagnosis of gemcitabine-induced lung injury. Discontinuation of gemcitabine and initiation of systemic steroids resulted in significant clinical improvement. This case highlights the diagnostic challenges of gemcitabine pulmonary toxicity in patients with metastatic lung disease and concurrent viral infection.

## Introduction

Gemcitabine is a nucleoside analog commonly used in the treatment of several solid malignancies, including pancreatic, lung, bladder, and renal cancers. Although generally well tolerated, pulmonary toxicity has been reported and may manifest as interstitial pneumonitis, diffuse alveolar damage, noncardiogenic pulmonary edema, or acute respiratory distress syndrome (ARDS) [1-3]. Severe pulmonary toxicity remains uncommon but may lead to significant morbidity and mortality when it occurs.

The exact mechanism of gemcitabine-induced lung injury remains incompletely understood. Proposed mechanisms include cytokine-mediated inflammatory responses, endothelial injury, and increased pulmonary capillary permeability leading to alveolar damage and impaired gas exchange [4,5]. Risk factors for pulmonary toxicity include combination chemotherapy with platinum agents, underlying lung disease, and extensive metastatic involvement of the lungs.

Renal medullary carcinoma is a rare and aggressive subtype of renal cell carcinoma characterized by loss of SMARCB1 (INI1) expression and a strong association with sickle cell trait. Patients often present with advanced metastatic disease involving the lungs, lymph nodes, liver, and other organs. Because of its aggressive nature, platinum-based chemotherapy regimens that include gemcitabine are frequently used for treatment [6].

Recognizing chemotherapy-related pulmonary toxicity in this population can be challenging because respiratory symptoms may mimic infection, tumor progression, pulmonary embolism, or cardiogenic pulmonary edema. Early recognition is important because prompt discontinuation of the offending agent and initiation of corticosteroids can result in rapid clinical improvement.

Here, we report a case of gemcitabine-induced lung injury presenting with acute hypoxic respiratory failure initially suspected to be multifocal pneumonia and evolving ARDS.

## Case presentation

A previously healthy woman in her 40s with metastatic SMARCB1-deficient renal medullary carcinoma presented with acute dyspnea, productive cough, and fever. She had no prior history of lung disease, was a lifelong non-smoker, and worked in an office setting without significant occupational or environmental exposures.

Her malignancy had been diagnosed a month before presentation, with metastatic disease involving the lungs, mediastinal lymph nodes, liver, and uterus. She was started on combination chemotherapy with gemcitabine and carboplatin and completed the second cycle two days before presentation.

Approximately one day after receiving the second chemotherapy dose, she developed rapidly progressive shortness of breath associated with cough and fever, prompting hospital presentation.

On arrival, she was tachypneic and hypoxic, requiring bilevel positive airway pressure (BiPAP) for respiratory support. As her respiratory status improved, she was transitioned to high-flow nasal cannula oxygen therapy.

Physical examination revealed diffuse bilateral crackles without peripheral edema or jugular venous distention. Laboratory evaluation demonstrated leukocytosis that later normalized, borderline anemia, mildly elevated but nondynamic BNP and troponin levels, and normal lactate. A multiplex viral respiratory panel detected rhinovirus, while blood and sputum cultures showed no bacterial growth (Table 1).

**Table 1:** Key laboratory results at presentation SARS-CoV-2: severe acute respiratory syndrome coronavirus 2, RSV: respiratory syncytial virus, MRSA: methicillin-resistant Staphylococcus aureus, PCR: polymerase chain reaction, IgM: immunoglobulin M, pCO₂: partial pressure of carbon dioxide, pO₂: partial pressure of oxygen, HCO₃⁻: bicarbonate

Test	Result	Reference range
Arterial blood gas		
pH	7.49	7.35-7.45
pCO₂	42 mmHg	35-45 mmHg
pO₂	301 mmHg	80-100 mmHg
HCO₃⁻	32 mmol/L	22-26 mmol/L
Base excess	7.9 mmol/L	-2 to +2 mmol/L
Oxygen saturation	97.9%	95%-100%
Hemoglobin (arterial)	9.9 g/dL	12-16 g/dL
Sodium (arterial sample)	131 mmol/L	135-145 mmol/L
Potassium (arterial sample)	3.8 mmol/L	3.5-5.0 mmol/L
Ionized calcium	1.16 mmol/L	1.12-1.32 mmol/L
Chloride (arterial sample)	97 mmol/L	98-107 mmol/L
Glucose (arterial sample)	141 mg/dL	70-110 mg/dL
Lactate (arterial sample)	1.0 mmol/L	0.5-2.0 mmol/L
Complete blood count		
White blood cell count	11.6×10⁹/L	4.0-10.5 ×10⁹/L
Hemoglobin	10 g/dL	12-16 g/dL
Hematocrit	30.7%	36%-48%
Mean corpuscular volume	76.6 fL	80-100 fL
Mean corpuscular hemoglobin	24.9 pg	27-33 pg
Mean corpuscular hemoglobin concentration	32.6 g/dL	31-36 g/dL
Red cell distribution width	17.4%	11%-15%
Platelet count	213×10⁹/L	150-400 ×10⁹/L
Absolute neutrophil count	11×10⁹/L	1.5-8.0×10⁹/L
Absolute lymphocyte count	0.37×10⁹/L	1-4×10⁹/L
Coagulation panel		
Prothrombin time	14.9 seconds	11-14 seconds
International normalized ratio	1.2	0.9-1.1
Chemistry panel		
Glucose	130 mg/dL	70-110 mg/dL
Blood urea nitrogen	18 mg/dL	7-20 mg/dL
Creatinine	0.6 mg/dL	0.6-1.2 mg/dL
Sodium	139 mmol/L	135-145 mmol/L
Potassium	4.1 mmol/L	3.5-5.0 mmol/L
Chloride	96 mmol/L	98-107 mmol/L
Carbon dioxide (serum bicarbonate)	26 mmol/L	22-29 mmol/L
Calcium	8.6 mg/dL	8.5-10.5 mg/dL
Albumin	3.1 g/dL	3.5-5.0 g/dL
Total protein	5.0 g/dL	6.0-8.3 g/dL
Aspartate aminotransferase	32 U/L	10-40 U/L
Alanine aminotransferase	31 U/L	10-55 U/L
Alkaline phosphatase	103 U/L	40-129 U/L
Cardiac markers		
Cardiac troponin	39 ng/L	<14 ng/L
B-type natriuretic peptide	4,059 pg/mL	<100 pg/mL
Inflammatory markers		
C-reactive protein	164 mg/L	<5 mg/L
Lactic acid	1.30 mmol/L	0.5-2.0 mmol/L
Infectious disease testing		
SARS-CoV-2 PCR	Not detected	Negative
Influenza A/B PCR	Not detected	Negative
RSV PCR	Not detected	Negative
MRSA nasal PCR	Not detected	Negative
Rhinovirus/enterovirus PCR	Detected	Negative
Mycoplasma IgM	<770 U/mL	0-769 U/mL

**Figure 1:**
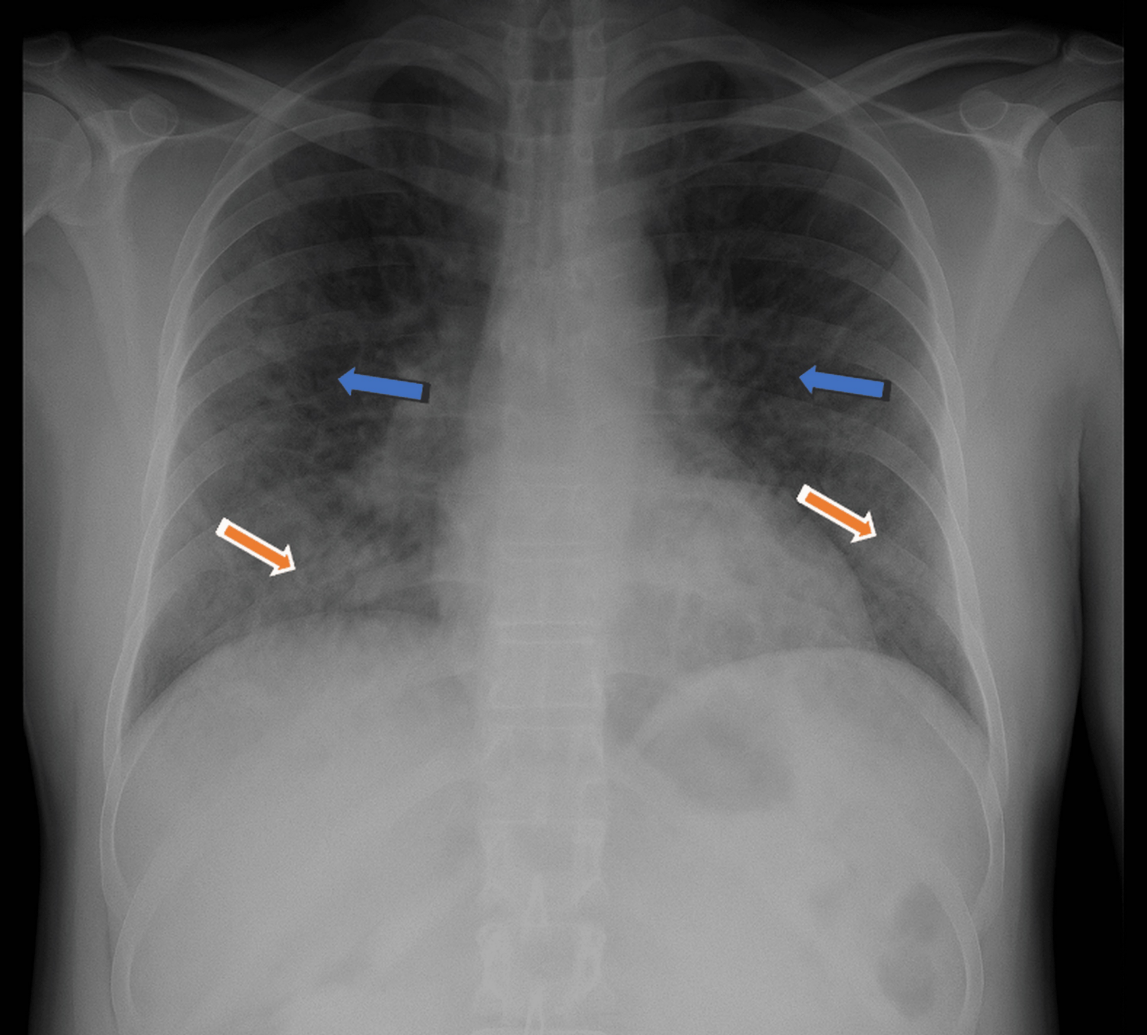
Chest X-ray showing diffuse bilateral interstitial (blue arrows) and airspace opacities (red arrows), more prominent in the lower lobes

**Figure 2:**
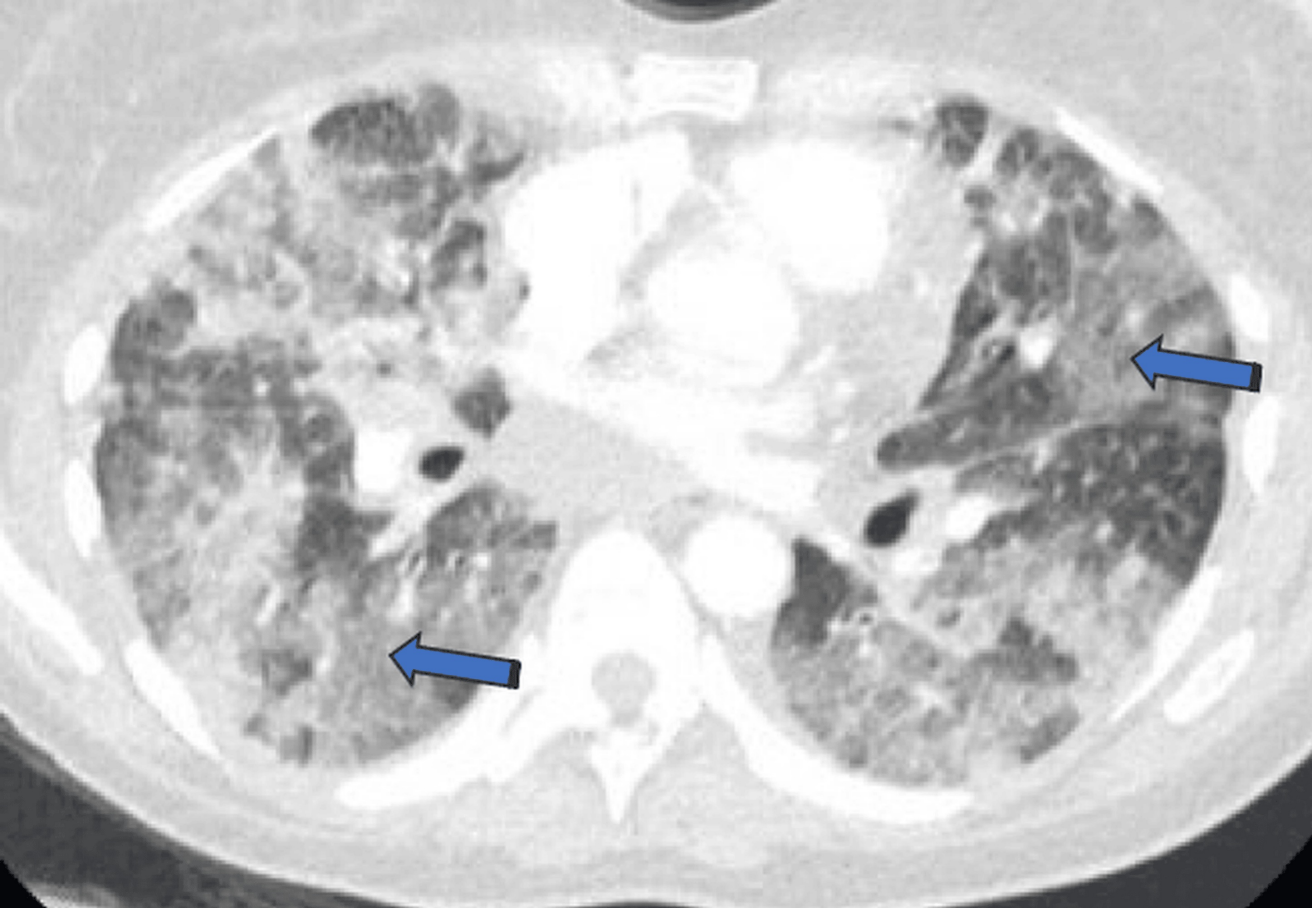
Computed tomography of the chest demonstrating diffuse lower lobe-predominant ground-glass opacities (blue arrows)

Chest radiography revealed diffuse bilateral interstitial and airspace opacities predominantly in the lower lung zones (Figure 1). Transthoracic echocardiography demonstrated preserved cardiac function without right ventricular dilation or pericardial effusion. Computed tomography angiography excluded pulmonary embolism and demonstrated diffuse lower lobe-predominant ground-glass opacities, patchy airspace involvement, peribronchial thickening, and a small right pleural effusion (Figure 2).

The patient was admitted to the intensive care unit for management of acute hypoxic respiratory failure. Empiric cefepime and azithromycin were initiated but discontinued once infectious etiologies were excluded.

Given the temporal association with chemotherapy and the absence of alternative etiologies, gemcitabine-induced lung injury was suspected. Intravenous methylprednisolone was initiated, resulting in gradual improvement in oxygenation and respiratory symptoms.

The patient remained in the intensive care unit for five days. During this time, she was transitioned from BiPAP to high-flow nasal cannula and subsequently to low-flow oxygen. Corticosteroid therapy was continued for a total of 10 days, beginning with high-dose intravenous methylprednisolone followed by tapering after five days with clinical improvement.

Gemcitabine therapy was discontinued. The patient continued to improve clinically and was transferred to the general medical floor.

She was discharged home after 10 days of hospitalization on 4 L of supplemental oxygen via nasal cannula. Steroids were discontinued after completion of the 10-day course without the need for a prolonged outpatient taper (Table 2).

**Table 2:** Timeline of clinical events and management ICU: intensive care unit, IV: intravenous, BiPAP: bilevel positive airway pressure

Timeline	Event
A month before presentation	Diagnosis of metastatic renal medullary carcinoma
2 days before presentation	Second cycle gemcitabine + carboplatin
Day 1 post-chemotherapy	Dyspnea, cough, fever
Hospital Day 1	BiPAP for acute hypoxic respiratory failure
Hospital Day 2	Transition to high-flow nasal cannula
Hospital Days 1-5	ICU admission
Hospital Day 2	IV methylprednisolone started
Hospital Day 6	Transfer to the medical floor
Hospital Day 10	Discharged on 4 L oxygen

## Discussion

Gemcitabine-induced pulmonary toxicity is an uncommon but clinically significant adverse effect, with presentations ranging from mild pneumonitis to rapidly progressive respiratory failure. Several mechanisms have been proposed, including immune-mediated hypersensitivity, cytokine-driven inflammation, endothelial injury, and gemcitabine-induced capillary leak [1-8]. The onset can occur early in the treatment course, often after one or two treatment cycles, consistent with this patient’s clinical presentation.

Diagnosis is challenging due to overlap with more common conditions such as bacterial or viral pneumonia, pulmonary embolism, disease progression, and cardiogenic pulmonary edema. In this case, metastatic lung disease and rhinovirus infection complicated the diagnostic process. Viral infections may potentiate chemotherapy-related lung injury by amplifying inflammatory pathways and increasing alveolar epithelial vulnerability [4,9,10].

Radiological findings in gemcitabine pulmonary toxicity commonly include diffuse ground-glass opacities, interstitial infiltrates, and lower lobe predominance, findings that were present in this patient. The preserved cardiac function and absence of pulmonary embolism supported a noncardiogenic etiology. The rapid clinical response to corticosteroids further supported the diagnosis, as steroid responsiveness is frequently reported in gemcitabine-associated lung injury [1-5,8-11].

Risk appears to be higher among patients receiving platinum-based combination chemotherapy, which may amplify pulmonary toxicity through synergistic endothelial injury or increased inflammatory cytokine release [5,6]. The presence of extensive pulmonary metastases in this patient may have further increased susceptibility by reducing pulmonary reserve and altering local immune responses.

In the present case, the patient required noninvasive ventilatory support followed by high-flow oxygen therapy and intensive care monitoring for several days, highlighting the potential severity of gemcitabine-associated pulmonary toxicity. Prompt recognition of the condition and early initiation of corticosteroid therapy resulted in rapid clinical improvement and prevented progression to invasive mechanical ventilation.

This case underscores the importance of maintaining a high index of suspicion for gemcitabine-induced lung injury in patients who develop respiratory symptoms shortly after chemotherapy administration.

Adverse drug reaction assessment

The adverse drug reaction observed in this case was evaluated using standard pharmacovigilance principles. Based on the Naranjo Adverse Drug Reaction Probability Scale, the association between gemcitabine exposure and the development of pulmonary toxicity in this patient is considered probable, given the clear temporal relationship with chemotherapy administration, improvement after discontinuation of the drug and initiation of corticosteroid therapy, and the absence of alternative etiologies such as bacterial infection, pulmonary embolism, or cardiogenic pulmonary edema [12-14].

The reaction can be classified as severe, as it resulted in acute hypoxic respiratory failure requiring noninvasive ventilatory support, high-flow oxygen therapy, and intensive care unit monitoring. Furthermore, the event meets the definition of a serious adverse drug reaction, since it required hospitalization and posed a potentially life-threatening condition.

Recognition of this adverse drug reaction allowed prompt discontinuation of gemcitabine and initiation of systemic corticosteroid therapy, which resulted in progressive clinical improvement and prevented further respiratory deterioration.

Clinical learning points

Gemcitabine-induced pulmonary toxicity is a rare but potentially life-threatening complication that may present with acute hypoxic respiratory failure and imaging findings mimicking pneumonia or acute respiratory distress syndrome.

Early recognition of chemotherapy-associated lung injury can be challenging in patients with metastatic cancer due to overlapping clinical features with infection, pulmonary embolism, or tumor progression.

Prompt discontinuation of the offending chemotherapeutic agent and initiation of corticosteroid therapy can result in rapid clinical improvement and prevent progression to invasive mechanical ventilation.

Clinicians should maintain a high index of suspicion for drug-induced pulmonary toxicity in patients who develop respiratory symptoms shortly after gemcitabine administration.

## Conclusions

Gemcitabine-induced lung injury should be considered in patients who develop new hypoxemia and diffuse pulmonary opacities after recent gemcitabine exposure, particularly when infectious, embolic, and cardiogenic etiologies have been excluded. Recognition of the temporal relationship between chemotherapy administration and symptom onset, along with characteristic imaging findings, is essential for timely diagnosis. Early discontinuation of gemcitabine and prompt initiation of corticosteroid therapy are critical interventions that may significantly improve patient outcomes.
